# IL-17A both initiates, via IFNγ suppression, and limits the pulmonary type-2 immune response to nematode infection

**DOI:** 10.1038/s41385-020-0318-2

**Published:** 2020-07-07

**Authors:** Jesuthas Ajendra, Alistair L. Chenery, James E. Parkinson, Brian H. K. Chan, Stella Pearson, Stefano A. P. Colombo, Louis Boon, Richard K. Grencis, Tara E. Sutherland, Judith E. Allen

**Affiliations:** 1 Lydia Becker Institute for Immunology & Infection, Faculty of Biology, Medicine & Health, Manchester Academic Health Science Centre, University of Manchester, Manchester, UK; 2 Wellcome Centre for Cell-Matrix Research, Manchester, M13 9PT UK; 3 Department of Tropical Disease Biology, Liverpool School of Tropical Medicine, Liverpool, L3 5QA UK; 4 Bioceros, Member of Polpharma Biologics, Yalelaan 46, 3584 CM Utrecht, The Netherlands

## Abstract

*Nippostrongylus brasiliensis* is a well-defined model of type-2 immunity but the early lung-migrating phase is dominated by innate IL-17A production. In this study, we confirm previous observations that *Il17a*-KO mice infected with *N. brasiliensis* exhibit an impaired type-2 immune response. Transcriptional profiling of the lung on day 2 of *N. brasiliensis* infection revealed an increased *Ifng* signature in *Il17a*-KO mice confirmed by enhanced IFNγ protein production in lung lymphocyte populations. Depletion of early IFNγ rescued type-2 immune responses in the *Il17a*-KO mice demonstrating that IL-17A-mediated suppression of IFNγ promotes type-2 immunity. Notably, later in infection, once the type-2 response was established, IL-17A limited the magnitude of the type-2 response. IL-17A regulation of type-2 immunity was lung-specific and infection with *Trichuris muris* revealed that IL-17A promotes a type-2 immune response in the lung even when infection is restricted to the intestine. Together our data reveal IL-17A as a major regulator of pulmonary type-2 immunity such that IL-17A supports early development of a protective type-2 response by suppression of IFNγ but subsequently limits excessive type-2 responses. A failure of this feedback loop may contribute to conditions such as severe asthma, characterised by combined elevation of IL-17 and type-2 cytokines.

## Introduction

Innate and adaptive sources of interleukin-17A (IL-17A) are responsible for a range of neutrophil-associated inflammatory conditions as well as protection from many bacterial and fungal pathogens.^1,2^ In contrast, type-2 immunity is required for effective control of most helminth infections^3^ and is characterized by eosinophilic inflammation and the cytokines IL-4, IL-5 and IL-13. When both type-2 and IL-17 responses are present during helminth infection enhanced pathology is observed, as shown for human schistosomiasis^4,5^ and onchocerciasis.^6^ The detrimental relationship between IL-17A and type-2 associated diseases has also been extensively documented in allergic asthma in which the most severe symptoms occur in patients with both high Th2 and Th17 cell responses.^7^ Critically, type-2 cytokines can actively suppress IL-17A production which may be an important feedback mechanism to avoid extreme IL-17A-driven pathology.^8–10^ Despite evidence for an important relationship between IL-17A and type-2 immune responses during chronic disease, how these responses are connected remain poorly understood.

We and others have demonstrated a prominent role for IL-17A during infection with the lung-migrating nematode *Nippostronglyus brasiliensis*,^8,11^ a well-defined pulmonary model of type-2 immunity. After entering the host via the skin, *N. brasiliensis* larvae migrate through the lung, causing tissue damage and haemorrhage. IL-17RA-dependent neutrophil recruitment is largely responsible for the lung damage in this model.^11^ We previously found that the chitinase-like protein Ym1 induces expansion of IL-17A-producing γδ T cells and Ym1 blockade or IL-17A-deficiency protects mice from peak lung damage.^8^ More surprising was our finding that Ym1 neutralisation or IL-17A-deficiency prevents the development of a full type-2 response during *N. brasiliensis* infection.^8^

The notion that IL-17A is required for development of a type-2 response appears counter to the evidence that type-2 cytokines suppress IL-17A production.^9,10^ However, previous studies using murine models of allergic inflammation also show impaired type-2 immunity in the face of IL-17A-deficiency^12,13^ or blockade.^14^ In an infection or injury context, the specific tissue as well as timing might all play decisive roles in whether IL-17A augments or suppresses type-2 responses. We therefore used *N. brasiliensis* infection to address the contribution of γδ T cell-derived IL-17A to the development of a subsequent type-2 immune response in the lung. We found that IL-17A suppressed early IFNγ production and that this suppression was essential for the optimal development of a type-2 response. Once the type-2 response was established, IL-17A acted as a negative regulator, revealing distinct roles during innate and adaptive stages of the response. Notably, *Trichuris muris*, a nematode restricted to the gastro-intestinal tract also induced a lung type-2 response that was IL-17A-dependent. However, we found no evidence that IL-17A regulated the intestinal type-2 response. Thus, IL-17A serves as a lung-specific regulator of the type-2 immune response.

## Results

### IL-17A-deficient mice mount a diminished type-2 response

**Fig. 1:**
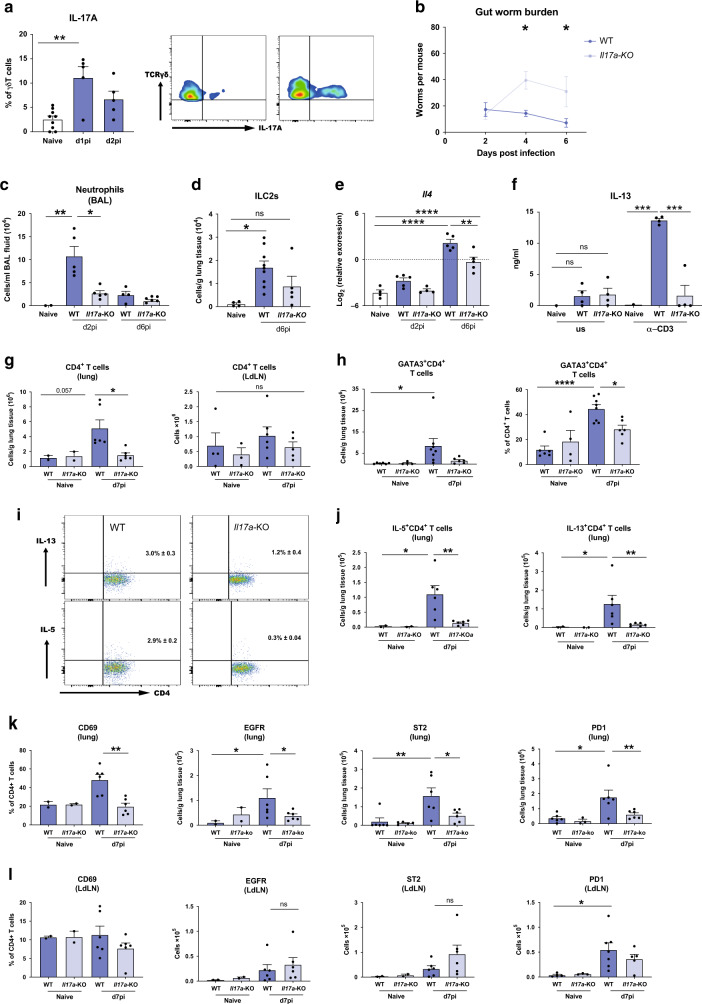
Mice deficient in IL-17A mount a diminished type-2 response at the site of infection. C57BL/6 (WT) and *Il17a-*KO mice were infected with 250 *N. brasiliensis* L3s and cell frequencies and cytokines were measured at different time points post infection compared with WT naïve mice. Frequencies of IL-17A-producing γδ T cells on d1pi and d2pi and representative flow-plot at d1pi (**a**). Worm burden in small intestine assessed in WT and *Il17a-*KO mice on days 2, 4 and 6 post *N. brasiliensis* infection (**b**). Absolute numbers of neutrophils (Ly6G^+^CD11b^+^) (**c**) and lung ILC2s (Lineage^−^ KLRG^+^CD127^+^CD90.2^+^ST2^+^) as measured via flow cytometry (**d**). Relative mRNA expression of cytokine *Il4* in whole lung as quantified by qRT-PCR (log2 expression relative to *actb* (β-actin)) (**e**). Secreted IL-13 levels from unstimulated (us) or 72 h α-CD3 treated single-suspension lung cells (**f**). Absolute numbers of live CD4^+^ T cells in lung tissue and lung draining lymph nodes (LdLN) (**g**). Frequency and absolute numbers of GATA3^+^ CD4^+^ T cells in the lung (**h**). Representative flow-plots showing the frequency of IL-5 and IL-13 production by CD4^+^ T cells d7pi in lung from WT and *Il17a-KO* infected mice (**i**). Absolute numbers of IL-5^+^ and IL-13^+^ CD4^+^ T cells in the lung (**j**). Expression of CD69 on CD4^+^T cells and absolute numbers of EGFR^+^CD4^+^T cells, ST2^+^CD4^+^T cells and PD-1^+^CD4^+^T cells in lung (**k**) and LdLNs (**l**). Data are representative (mean ± s.e.m.) of at least 3 individual experiments (**a**, **c**–**l**) or pooled data from three experiments (**b**). Data were tested for normality using Shapiro-Wilk test and analysed using one-way ANOVA followed by Sidak’s multiple comparisons test for selected groups. NS – not significant. Data in (**e**) were log2 transformed to achieve normal distribution and statistical tests were performed on transformed data *P < 0.05, **P < 0.01, ***P < 0.001, ****P < 0.0001.

In keeping with the known ability of *N. brasiliensis* to induce a strong pulmonary type-2 immune response on day 6 post infection (d6pi), we found the bronchoalveolar lavage (BAL) and lungs of C57BL/6 mice to be dominated by eosinophils (Supplementary Fig. 1a, b). This response was accompanied by elevated numbers of CD4^+^ T cells as well as induction of Group 2 Innate lymphoid cells (ILC2, Supplementary Fig. 1c, d). The establishment of a type-2 response was further confirmed by increased type-2 cytokine expression by CD4^+^ T cells and gene expression in whole lung (Supplementary Fig. 1e, f). As we and others previously reported,^8,11^ infected mice exhibited increased IL-17A production within the first 48 h post infection (Fig. 1a) and consistent with previous reports, the main source of IL-17A was γδ T cells.^8^ On d2pi the BAL consisted mainly of neutrophils (Supplementary Fig. 1a), which, together with *N. brasiliensis* larvae migration, is known to cause acute lung injury.^11^

To investigate the role of IL-17A during the development of type-2 immune responses, we infected *Il17a-*KO mice and WT C57BL/6 controls with *N. brasiliensis* L3’s. Larvae leave the lung within 48 h and are expelled from the gut within 6–8 days. Consistent with our previous findings^8^ *Il17a-*KO mice were significantly more susceptible to infection exhibiting an intestinal worm burden almost two-fold higher than WT controls on d4pi and d6pi (Fig. 1b). As expected, the early d2 neutrophilia in response to *N. brasiliensis* infection was muted in the *Il17a-*KO mice relative to the WT controls (Fig. 1c). Between d2pi and d6pi, there was a switch from neutrophilic to eosinophilic responses in the lungs (Supplementary Fig. 1a). Whilst increased eosinophil numbers were observed in all d6 infected mice relative to naïve animals, this increase was less evident for *Il17a-*KO mice (Supplementary Fig. 1g). ILC2s displayed a similar pattern, as cell numbers significantly increased with infection in WT but not in *Il17a-KO* mice (Fig. 1d). In addition, increased expression of the hallmark type-2 cytokine *Il4* was observed at d6pi in WT mice, whilst *Il17a-*KO mice had significantly reduced *Il4* expression compared with WT controls (Fig. 1e). Secretion of IL-13 protein levels at d6pi by total lung cells restimulated with α-CD3 were significantly higher in WT mice compared with naïve controls, whilst *Il17a-*KO mice failed to secrete detectable amounts of IL-13 (Fig. 1f). We also measured expression levels of the major mucins in the lung because host mucin production is another feature of protective type-2 responses.^15^ *N. brasiliensis* infection drove an early increase in mucins *Muc5ac* and *Muc5b* expression in the lungs of WT mice at d2pi corresponding to a timepoint when the larvae are transitioning from the lungs. Expression of both *Muc5ac* and *Muc5b* were increased in WT mice compared with naïve controls, but reduced in *Il17a-*KO mice (Supplementary Fig. 1h).

While we observed an impairment in innate type-2 features, we next aimed to determine whether the impact of IL-17A-deficiency on type-2 immunity was due to changes in the adaptive immune response, mainly T cell activation or polarisation during *N. brasiliensis* infection. Using flow cytometry, we observed a failure to induce CD4^+^ T cells numbers on d7pi in the lungs in *Il17a-*KO mice compared with WT controls (Fig. 1g). In contrast, there were no significant differences in CD4^+^ T cell numbers in the lung-draining lymph nodes (Fig. 1g). We also examined expression of the Th2 transcription factor GATA3. While WT mice showed a significant increase in absolute numbers and frequency of GATA3^+^CD4^+^ T cells upon infection, *Il17a-*KO mice had a significantly lower frequency of GATA3^+^CD4^+^ T cells and failed to upregulate these cells on d7pi compared with WT controls (Fig. 1h). Not only were there fewer GATA3^+^ CD4^+^ T cells in the lungs of *Il17a-*KO mice, the CD4^+^ T cells in *Il17a*-KO mice produced significantly less IL-13 and IL-5 (Fig. 1i). Strikingly, by d7pi, IL-5^+^ and IL-13^+^ CD4^+^ T cell numbers did not increase in *Il17a*-KO mice in response to infection (Fig. 1j). At the same time point post-infection, we also found that expression of the activation marker CD69 was upregulated on CD4^+^ T cells in the lungs of WT but not *Il17a-*KO mice (Fig. 1k). However, CD69 did not differ between all tested groups in the lung-draining lymph nodes (Fig. 1l). Recently, Minutti et al. showed that epidermal growth factor receptor (EGFR) in complex with ST2 on T cells allows for IL-33-induced IL-13 production at the site of *N. brasiliensis* infection.^16^ We therefore analysed surface expression of EGFR and ST2 on lung and lung-draining lymph node T cells. *N. brasiliensis* infection increased the number of CD4^+^ T cells expressing these markers in the lung, but this increase was significantly reduced in *Il17a-*KO mice. (Fig. 1k). No significant changes to ST2 and EGFR expression between WT and KO mice were observed in the lung-draining-lymph nodes (Fig. 1l). We also measured PD-1 expression, an important regulator of T cell function during helminth infection.^17,18^ *N. brasiliensis* infection in WT mice led to increased numbers of CD4^+^ T cells expressing PD-1 in the lung and the lung draining lymph node of WT mice (Fig. 1k, l). However, there were significantly fewer PD-1^+^ CD4^+^ T cells in the lungs (Fig. 1k) of infected *Il17a-*KO mice but not the lymph node (Fig. 1l). Overall, these data demonstrated a significant impairment of the type-2 response in the absence of IL-17A during helminth infection, with lung CD4^+^ T cells failing to become fully activated and produce type-2 cytokines.

### IL-17A leads to a downregulation of early IFNγ during *Nippostronglyus* infection

**Fig. 2:**
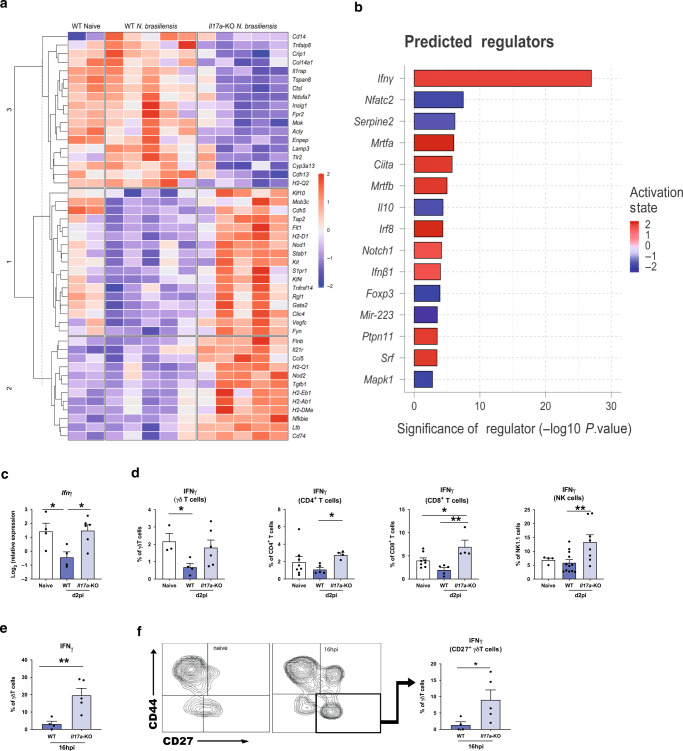
Presence of IL-17A leads to a downregulation of early IFNγ during *N. brasiliensis* infection. Whole lung RNA from C57BL/6 (WT) and *Il17a*-KO mice on d2pi with *N. brasiliensis* compared with WT naïve mice, were analysed by Nanostring. Unsupervised, hierarchically clustered heat map showing significant differentially expressed genes between infected WT, *Il17a-*KO mice and uninfected (naïve) WT (**a**). Top differentially regulated genes from (a) between infected WT and *Il17a-*KO mice were run in Ingenuity pathway analyzer, with top predicted regulators shown in (**b**). Relative expression of *Ifng* in whole lung of naïve WT and d2 *N. brasiliensis* infected WT and *Il17a-*KO mice (log2 expression relative to *actb* (β-actin)) (**c**). Frequencies of IFNγ^+^ γδ T cells, CD4^+^ T cells, CD8^+^ T cells and NK cells in WT and *Il17a-*KO mice d2pi compared with WT naïve mice as assessed by flow cytometry (**d**). Frequency of IFNγ^+^ γδ T cells 16 h post *N. brasiliensis* infection in WT and *Il17a-*KO mice (**e**). Representative flow plots showing CD44 and CD27 γδ T cell subsets in naïve mice and mice 16 h post *N. brasiliensis* infection as well as frequency of IFNγ^+^ CD27^+^ γδ T cells 16 h post *N. brasiliensis* infection in WT and *Il17a-*KO mice (**f**). Data (**c**–**f**) are expressed as mean ± s.e.m. and are representative of at least 2 individual experiments with at least three mice per infected group. Data were tested for normality using Shapiro-Wilk test and analysed using one-way ANOVA followed by Sidak’s multiple comparisons test for selected groups or student’s *t* test. *P < 0.05, **P < 0.01.

Rapid early IL-17A production is critical for protective immune responses in different settings of lung immunity.^1,19^ To better understand the early events unfolding in the lung during *N. brasiliensis* infection, we performed a Nanostring gene expression array using a myeloid immunity panel (700 genes). In whole lung, differentially expressed (DE) genes between naïve WT mice and infected WT and *Il17a-*KO mice at d2pi were assessed in total unamplified RNA (Fig. 2a). IL-17A deficiency led to a distinct gene expression profile compared with WT mice in response to *N. brasiliensis* infection. Notably, when analysing all DE genes (Fig. 2a) using the Ingenuity pathway analyser (Qiagen), *ifng* was predicted as the most significantly increased upstream regulator in *N. brasiliensis* infected *Il17a-*KO compared with WT mice (Fig. 2b). This led us to hypothesize that IL-17A may suppress IFNγ, which would facilitate Th2 cell development and explain why mice deficient in IL-17A cannot induce a full type-2 immune response. This hypothesis was also consistent with our unpublished and published^20^ finding that Ym1, which induces IL-17A, strongly suppresses IFNγ. To test this possibility, we assessed IFNγ responses in *Il17a-*KO mice after *N. brasiliensis* infection. While WT mice exhibited significant suppression of *Ifng* in whole lung at 2dpi compared with uninfected controls, mice deficient in IL-17A did not show this phenotype (Fig. 2c). By intracellularly staining for IFNγ, we observed that *Il17a-*KO mice infected with *N. brasiliensis* failed to exhibit the early downregulation of IFNγ expression seen in infected WT mice (Fig. 2d). Importantly, this failure of suppression was observed across different types of IFNγ-producing cells. Lung γδ T cells, CD4^+^ T cells, CD8^+^ T cells as well as NK1.1^+^ natural killer cells from *Il17a-*KO mice all produced significantly enhanced IFNγ relative to WT mice (Fig. 2d). We further assessed whether early IFNγ was produced by γδ T cell subsets that differ in their expression of CD27 and CD44.^21^ At 16 h post *N. brasiliensis* infection, IFNγ frequencies were also significantly increased in γδ T cells of *Il17a-*KO mice compared with WT controls (Fig. 2e). Consistent with expectations,^21^ the CD27^+^ γδ T cells were the main producers of IFNγ after infection (Fig. 2f). Overall, our data demonstrated that IL-17A-deficiency enhanced IFNγ production during infection, supporting the hypothesis that IL-17A plays an important role in downregulating IFNγ at the site of infection during the lung migratory phase of *N. brasiliensis* infection.

### IFNγ neutralization in *Il17a*-KO mice rescues the impaired type-2 immune response

**Fig. 3:**
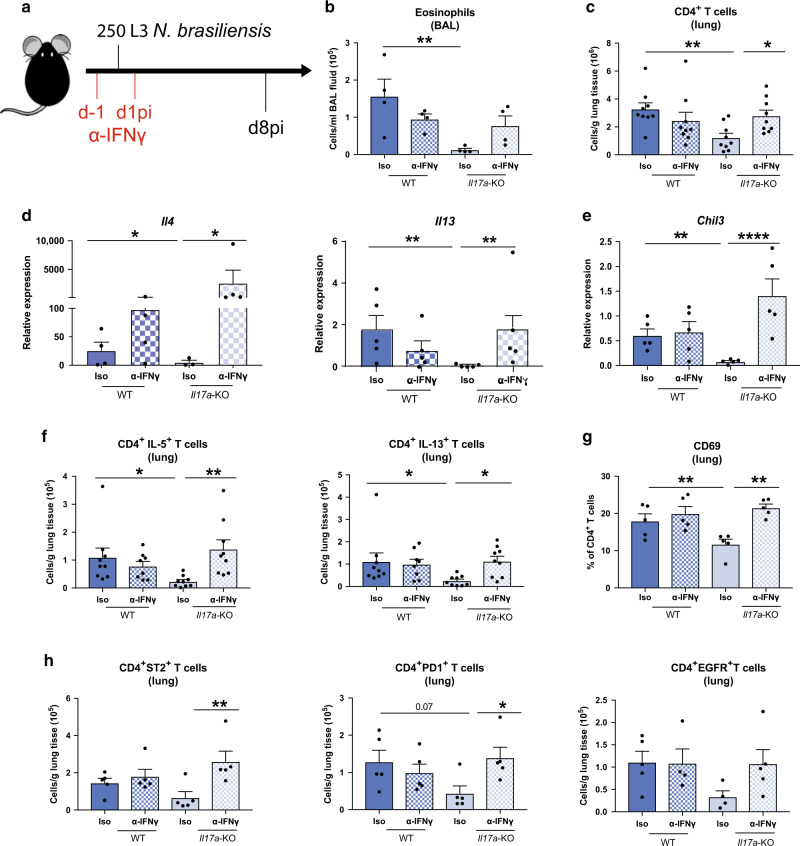
IFNγ neutralization in *Il17a-*KO mice rescues the impaired type-2 immune response. C57BL/6 (WT) and *Il17a-*KO mice and were treated with α-IFNγ or isotype control on days −1 and 1pi with 250 L3 larvae of *N. brasiliensis* (**a**). Absolute numbers of eosinophils per mL of BAL (**b**) or CD4^+^ T cells per gram lung tissue (**c**) as measured via flow cytometry on d8pi. Relative mRNA expression of type-2 cytokines *Il4* and *Il13* (**d**) and type-2 marker *Chil3* (**e**) from whole lung. Absolute numbers of IL-5^+^ and IL-13^+^ CD4^+^ T cells (**f**). Frequency of CD69^+^ CD4^+^ T cells (**g**) and numbers of EGFR^+^, ST2^+^ and PD1^+^ CD4^+^ T cells per gram lung tissue (**h**). Data (**b**, **d**, **e**–**g**) are representative (mean ± s.e.m.) of two individual experiments with at least three mice per group (per experiment) or pooled data from two experiments (**c**, **f**). Data were tested for normality using Shapiro-Wilk test and analysed using one-way ANOVA followed by Sidak’s multiple comparisons test for selected groups. Data in (**d**, **e**) were log2 transformed to achieve normal distribution and statistical tests were performed on transformed data. *P < 0.05, **P < 0.01, ***P < 0.001.

We next asked whether global suppression of early IFNγ by IL-17A was required for the full development of type-2 immunity in the lung. IFNγ was neutralised at day −1 and 1 of infection in *Il17a-*KO and WT mice, and responses examined at d8pi, a time point when the type-2 response should be fully developed (Fig. 3a). The significant defect in eosinophilic responses in *Il17a*-KO mice compared with WT mice was still evident at d8pi. However, blocking IFNγ in *Il17a*-KO mice enhanced eosinophil numbers (Fig. 3b). The same pattern was observed for numbers of CD4^+^ T cells in the lungs (Fig. 3c). To determine whether IFNγ neutralisation altered the type-2 response, we assessed expression of the key type-2 cytokines *Il4* and *Il13* and the type-2 marker *Chil3*. As expected, based on our results thus far, expression of these genes in the lungs was significantly reduced in infected *Il17a-*KO mice compared with WT mice (Fig. 3d, e). Notably, IFNγ depletion completely recovered expression of these cytokines in *Il17a*-KO mice compared with isotype-treated animals, with *Il4* expression surpassing the levels seen in WT infected mice (Fig. 3d). Similarly, analysis of numbers of IL-5 and IL-13 producing CD4^+^ T cells at d8pi showed complete restoration of the type-2 response in *Il17a-*KO mice that received the neutralising IFNγ antibody (Fig. 3f). Consistent with the ability of IFNγ to regulate type-2 cytokines, IFNγ depletion also restored the activation status of CD4^+^ T cells in the *Il17a-*KO mice as shown by CD69 expression (Fig. 3g) and increased the numbers of CD4^+^ T cells expressing type-2 markers EGFR, PD1 and ST2 (Fig. 3h). Again, no effect was observed in these parameters in IFNγ-depleted WT mice. Together these data demonstrate that an initial reduction in IFNγ levels during *N. brasiliensis* infection mediated by IL-17A, allows the subsequent development of type-2 immunity in the lung.

### IL-17A suppresses an established type-2 response in the lung

**Fig. 4:**
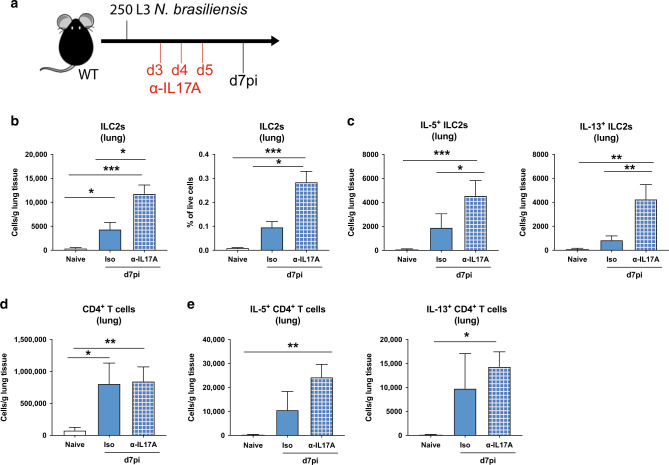
Late stage IL-17A suppresses type-2 immune responses during *N.brasiliensis* infection. C57BL/6 WT mice were treated with α-IL-17A or isotype control on days 3, 4, and 5pi with 250 L3 *N. brasiliensis* and responses measured at d7pi compared with uninfected (naïve) mice (**a**). Cells per gram lung tissue and frequency of ILC2s in live lung cells (**b**). Absolute number of IL-5^+^ and IL-13^+^ ILC2s per gram lung tissue (*n* = 5 for naïve, *n* = 11–12 for d7 *N. brasiliensis* infected groups) (**c**). Absolute numbers of CD4^+^ T cells (**d**) and IL-5^+^ and IL-13^+^ CD4^+^ T cells per gram of lung tissue (*n* = 3 for naïve and *n* = 6 for d7 *N. brasiliensis* infected groups) (**e**). Data pooled from two independent experiments (**b**, **c**) or are representative (mean ± s.e.m.) of 3 individual experiments with at least 3 mice per group (per experiment) (**d**, **e**). Data were tested for normality using Shapiro-Wilk test and analysed by a one-way ANOVA followed by Sidak’s multiple comparisons test for selected groups. *P < 0.05, **P < 0.01, ***P < 0.001.

IFNγ depletion in *Il17a-*KO mice not only restored the type-2 response, but in some cases exceeded WT levels. Therefore, we hypothesized that although innate IL-17A promotes the establishment of type-2 immunity in *N. brasiliensis* infection, once the adaptive response is in place, IL-17 can act to negatively regulate the type-2 pulmonary response. To test this hypothesis, we neutralized IL-17A at d4pi, d5pi and d6pi in WT mice and assessed immune responses at d7pi (Fig. 4a). Blocking of later stage IL-17A led to a significant increase in both ILC2 numbers and frequencies in the lung (Fig. 4b), as well as the numbers of ILC2s producing IL-5 and IL-13 (Fig. 4c). Although CD4^+^ T cell numbers in the lung were comparable between isotype-treated and anti-IL-17A-treated WT mice (Fig. 4d), the ability of CD4^+^ T cells to produce type-2 cytokines may partly rely on IL-17A, as mice administered anti-IL-17A showed a slight increase in numbers of CD4^+^ T cells producing IL-5 and IL-13 (Fig. 4e). This data demonstrated that IL-17A can have differential effects depending on the time and status of infection. While early IL-17A promotes the type-2 response, later in infection IL-17A acts to suppress and limit excessive type-2 immunity, particularly in ILC2s.

### IL-17A does not regulate type-2 immune responses at the site of *T. muris* infection

**Fig. 5:**
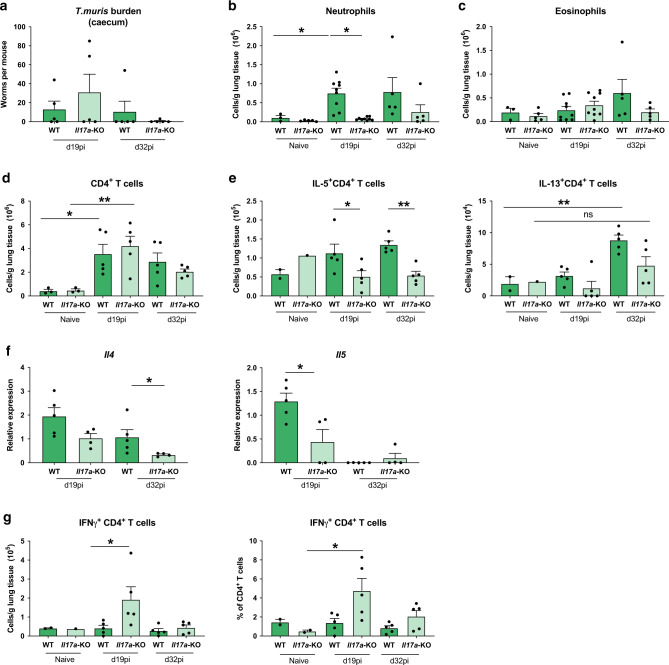
Lack of IL-17A impairs concurrent type-2 immune responses in the lung following infection with *Trichuris muris*. C57BL/6 WT and *Il17a*-KO mice were infected with a high dose of *T. muris* and immune parameters investigated at d19 and d32pi compared with uninfected (naïve) C57BL/6 WT and *Il17a*-KO mice. Worms counts in the caecum (**a**). Absolute numbers of neutrophils (**b**), eosinophils (**c**) and CD4^+^ T cells (**d**) per gram of lung at d19pi and d32pi compared with naïve mice. Absolute numbers of IL-5^+^ and IL-13^+^ CD4 + T cells per gram of lung tissue (**e**). Relative mRNA expression of cytokines *Il4* and *Il5* from whole lung (log2 expression relative to *actb* (β-actin)) of infected mice (**f**). Absolute numbers and frequency of IFNγ^+^ CD4^+^ T cells per gram of lung tissue (**g**). Data are expressed as mean ± s.e.m. and are representative of 3 individual experiments with at least four mice per infected group and one mouse per control group. Data were tested for normality using Shapiro-Wilk test and analysed with one-way ANOVA followed by Sidak’s multiple comparisons test for selected groups. Data in (**f**) were log2 transformed to achieve normal distribution and statistical tests were performed on transformed data *P < 0.05, **P < 0.01.

Our data demonstrate an impairment of the type-2 immune response in the lung during infection of *Il17a-*KO mice with the lung-migrating nematode *N. brasiliensis*. We wanted to investigate whether impairment of type-2 immunity by IL-17A was unique to the pulmonary environment. Initially we examined type-2 cytokine gene expression in the small intestine of *N. brasiliensis* infected mice at d7pi. However, we did not observe any significant changes in WT infected mice (data not shown). We therefore decided to use *Trichuris muris*, a nematode that establishes infection solely in the gastro-intestinal tract. Infection with *T. muris* begins with the ingestion of infective eggs that accumulate in the caecum. L1 larvae hatch and penetrate the caecum and proximal colon wall, undergoing moults to L2 (d9-11pi), L3 (d17pi), L4 (d22 pi) and adults (d29-32). High dose infection in C57BL/6 mice induces a strong type-2 response by d17pi, and subsequent clearance of the adult parasites.^22,23^ We infected WT and *Il17a-*KO mice with a high dose of 200 *T. muris* eggs and found worm counts in the caecum were comparable between mouse strains at d19pi and d32pi (Fig. 5a), indicating IL-17A does not alter parasite expulsion rate. Cell numbers in the caecum were analysed and no differences in eosinophil and neutrophil frequency were observed between *Il17a-*KO mice and WT controls on d19pi and d32pi (Supplementary Fig. 2a, b), suggesting an IL-17A-independent recruitment mechanism for both these cell types in the large intestine. CD4^+^ T cell numbers in the mLN were also comparable on d19pi and d32pi between *Il17a-*KO mice and WT controls (Supplementary Fig. 2c). Although there was an induction of type-2 cytokines in infected mice as measured by intracellular cytokine staining, the numbers of IL-4, IL-5 or IL-13-producing CD4^+^ T cells in the mLNs did not significantly differ between the groups (Supplementary Fig. 2d). Similarly, the relative expression of cytokines *Il4*, *Il5* and *Il13* did not differ between *Il17a-*KO mice and WT controls within the caecum (Supplementary Fig. 2e). Secreted levels of IL-5, IL-9 and IL-13 in MLN cells were also not impaired in *Il17a-*KO mice relative to WT controls (Supplementary Fig. 2f). Together, these data failed to provide any evidence that IL-17A was an important regulator of type-2 immunity in the intestine or draining lymph nodes during *T. muris* infection.

Previous studies have shown that despite the restriction of the *T. muris* lifecycle to the gastro-intestinal tract of the mammalian host, evidence of a type-2 immune response can be observed at distant sites, such as the lung.^24^ Therefore, the immune response in the lung of *T. muris* infected WT vs *Il17a-*KO mice at d19pi and d32pi was assessed. Neutrophils numbers were increased in infected WT animals at d19pi and d32pi, but this was significantly reduced in *Il17a-*KO mice on d19pi (Fig. 5b). No significant changes were observed for eosinophils (Fig. 5c). Whilst lung CD4^+^ T cell numbers in infected animals did not change compared with naïve controls (Fig. 5d), *Il17a*-KO mice had significantly fewer IL-5^+^CD4^+^ T cells at d19 and d32pi compared with WT controls (Fig. 5e). Although the effect on IL-13^+^CD4^+^ T cells was less evident, infected *Il17a*-KO mice failed to significantly increase numbers of IL-13^+^CD4^+^ T cells compared with uninfected controls (Fig. 5e). Supporting the intracellular cytokine staining, qRT-PCR analysis in whole lung tissue showed an impairment of type-2 cytokines in the *Il17a-*KO mice, with significantly decreased expression of *Il4* (d32pi) and *Il5* (d19pi) (Fig. 5f).

Similar to infection with *N. brasiliensis*, we also observed an upregulation of IFNγ in the lung during *T. muris* infection in *Il17a-*KO mice. Both the number and the frequency of IFNγ^+^CD4^+^ T cells in the lung were significantly increased in *Il17a-*KO compared with WT infected mice on d19pi (Fig. 5g). This data utilising *T. muris* infection models suggests that IL-17A-dependent suppression of IFNγ allows promotion of the type-2 immune response specifically in the lungs but not the intestine and highlights means of communication between the intestine and the lung involving IL-17A, not previously described.

## Discussion

IL-17A, the key cytokine of the IL-17 family, is central to barrier immunity, combating fungal infections and inducing antimicrobial proteins as well as neutrophil activating and recruiting chemokines.^2^ However, in the context of type-2 immunity, a combination of type-2 cytokines and IL-17A is often a signature for severe disease pathology. For example, IL-17A contributes to asthma pathology by enhancing IL-13 activity^25^ and a dysregulated balance between IL-17A and type-2 responses exacerbates pathology during schistosomiasis and onchocerciasis.^4–6,26^ Understanding the relationship between IL-17A and type-2 immune responses is thus critical, and we and others have previously demonstrated that development of a full type-2 response can require IL-17A.^8,12,13,27^

In our effort to understand how IL-17A might be required for full type-2 immunity, we have discovered that IL-17A suppresses early IFNγ expression in the lung during helminth infection. Although several studies show links between IL-17A and IFNγ, whether IFNγ is up- or downregulated in response to IL-17A varies with setting, timing and location. For example enhanced IFNγ in *Il17a-*KO mice has been described in a viral infection,^28^ experimental visceral leishmaniasis, and *Toxoplasma gondii* infection.^29^ Evidence also exists in the context of helminth infection, where a lack of IL-17A drives elevated IFNγ during infection with the filarial nematode *Litomosoides sigmondontis*^30^ or *Schistosoma japonicum*^31^ and *Schistosoma mansoni*.^5^ In contrast, IL-17A can promote IFNγ production during kidney-ischaemic reperfusion injury,^32^ or *Francisella tularensis* infection.^33^ Importantly, the consequence of IL-17A-IFNγ cross-regulations in the context of type-2 inflammation has never been shown and here we reveal IFNγ downregulation as a new mechanism through which IL-17A establishes a protective type-2 response in the lung.

Another key finding of our study was that the requirement for IL-17A to suppress IFNγ appears restricted to the lung. The protective type-2 immune response in the gut of *Il17a-*KO mice was not impaired, and mice were still able to expel *N. brasiliensis* from the small intestine and *T. muris* from the colon. The more surprising finding was that even though *T. muris* does not have a lung stage, the concurrent type-2 response in the lung was impaired in *Il17a-*KO mice. CD4^+^ T cells in the lung produced less type-2 cytokines in the *Il17a-*KO mice and consistent with our findings in *N. brasiliensis*, CD4^+^ T cells in *Il17a-KO* mice produced significantly higher amounts of IFNγ than in their WT counterparts. These findings raise major questions as to the nature of the insult that induces IL-17 in the lungs of *T. muris* infected mice. Although our data suggest that the impact of IL-17A on type-2 development might be lung restricted, there may still be a fundamental requirement for suppression of IFNγ for type-2 immunity to progress. Artis et al. demonstrated that the type-2 immune response during *T. muris* requires TSLP, and in very similar experiments to those described here, demonstrated that TSLP functions to suppress IFNγ.^34^ Thus, early suppression of IFNγ may be a general pre-requisite for the development of a type-2 environment with a requirement for IL-17A in the lung and TSLP (or other factors) in the gut.

We have not yet addressed the full mechanism behind IL-17A-mediated suppression of IFNγ during *N. brasiliensis* infection but it is notable that IL-17A not only impairs type-2 cytokine production, but also alters the cellular activation status and expression of type-2 markers. Interestingly, in our model, we only observe impairment of type-2 immune responses in the lung itself and not in the Th2 cells from lung-draining lymph nodes. Expression of EGFR and ST2, two markers closely associated with type-2 settings,^16^ were reduced on the CD4^+^ T cells of *Il17a-*KO mice in the lung. EGFR expression on Th2 cells is critical for resistance during GI helminth infection and a signalling complex between EGFR and ST2 can activate Th2 cells to secrete IL-13 in an antigen-dependent manner upon IL-33 exposure. Our data would suggest that this “licensing” of Th2 cells does not occur in the *Il17a-*KO mice during *N. brasiliensis* infection, indicating that IL-17A is needed for a proper induction of the adaptive Th2 response in the lung.

It is well documented that type-2 responses are essential to limit excessive IL-17^35–37^ but a novel finding from our study is that the reverse is also true. While the early γδ T cell-derived IL-17A supported the type-2 response, late IL-17A, derived from both Th17 cells and γδ T cells, negatively regulated type-2 cytokines. To our knowledge, IL-17A suppression of type-2 cytokines has not previously been described in vivo and illustrates a major cross-regulatory axis between type-2 cytokines and IL-17A, each required to contain the excessive production of the other. In the numerous situations in which a combination of type-2 cytokines and IL-17A results in severe disease pathology,^4,6,25^ it is apparent that this cytokine balance has failed. Together our data demonstrate that early events in the lung shape the protective type-2 immune response, with IL-17A as a critical regulator of type-2 immunity. IL-17A, as a driver of tissue damage,^8^ may itself be needed to establish a subsequent type-2 repair response. However, the ability of IL-17A to then suppress type-2 responses, reveal an important feedback loop that must go awry during severe asthma and other type-2 conditions in which IL-17A plays a damaging and pathogenic role. Finally, in combination with previous data,^34^ suppression of IFNγ at barrier sites may be a central paradigm for type-2 immunity.

## Materials and methods

### Mice and ethics statement

For experiments using only WT mice, C57BL/6 J mice were obtained from Charles River. C57BL/6 *Il17a*^Cre^*Rosa26*^eYFP^ mice were originally provided by Dr Brigitta Stockinger.^38,39^ For *Il17a-*KO experiments C57BL/6 WT mice and C57BL/6 *Il17a*^Cre^*Rosa26*^eYFP^ homozygote mice were bred at the University of Manchester. Mice were age- and sex-matched and all mice were housed in individually ventilated cages. Both males and females were used. Mice were not randomized in cages, but each cage was randomly assigned to a treatment group. Mice were culled by asphyxiation in a rising concentration of CO_2_. Experiments were performed in accordance with the United Kingdom Animals (Scientific Procedures) Act of 1986.

### *N. brasiliensis* infection

*N. brasiliensis* was maintained by serial passage through Sprague-Dawley rats, as described.^40^ Third-stage larvae (L3) were washed ten times with PBS (Dulbecco’s PBS, Sigma) before infection. On day 0, mice were infected subcutaneously with 250 or 500 larvae (L3). At various time points mice were euthanised, BAL was performed with PBS containing 1% BSA and lungs were taken for further analysis. For worm counts, the small intestines of infected mice were collected in PBS. Small intestines were then cut longitudinally along the entire length, placed in a 50 ml Falcon and incubated at 37 °C for 4 h. Settled worms were then counted with the aid of a dissecting microscope.

### Flow cytometry

Single-cell suspensions of the lung were prepared by digesting minced lung lobes for 30 min at 37 °C with 0.2 U/ml Liberase TL (Roche) and 80 U/ml DNase (Life Tech) in Hank’s balanced-salt solution before forcing tissue suspensions through a cell strainer (70 µm, Greiner). Red blood cells were lysed using Red Blood Cell Lysing Buffer Hybri Max (Sigma) for 3 min at RT and reaction was stopped by diluting samples in PBS. Total live cells were counted with AO/PI dye on an automated cell counter (Auto2000, Nexcelom). Cells were stained for live/dead (Life Technologies) and then incubated with Fc-block (1:500 CD16/CD32 and 1:50 mouse serum) and were then stained with fluorescence-conjugated antibodies. Cells were identified by expression of surface markers as follows: neutrophils Ly6G^+^CD11b^+^, eosinophils CD11b^+^ CD11c^−^ SigF^+^, CD4 T cells CD4^+^, TCRβ^+^CD11b^−^, γδ Tcells TCRβ^−^, TCRγδ^+^, CD11b^−^ and ILC2s Lineage^−^ (CD11b, TCRβ, TCRγδ, Ly6G, F4/80, CD11c, SigF, CD19) CD90.2^+^KLRG^+^CD127^+^. Antibody clones used are listed in Table 1. For staining of intracellular cytokines, cells were stimulated for 4 h at 37 °C with cell stimulation cocktail containing protein transport inhibitor (eBioscience), then stained with live/dead. After surface antibody staining, cells were fixed for 10 min at 4 °C using IC fix (Biolegend) and cells were then incubated in for 20 min at RT in Permeabilization buffer (biolegend). Intracellular staining was performed for cytokines using antibodies for IL-5, IL-13, IL-17A and IFNγ as well as for Gata3 and Ym1. Samples were analysed by flow cytometry with LSR Fortessa or LSR II (Becton-Dickinson) and data analysed using FlowJo v10 software.

**Table 1:** List of flow cytometry antibodies used.

Antigen	Clone	Manufacturer
CD11b	M1/70	BioLegend
CD11c	N418	BioLegend
Ly6C	HK1.4	BioLegend
CD4	GK1.5	BioLegend
F4/80	BM8	eBioscience
CD90.2	30-H12	Biolegend
CD127	A7R34	Invitrogen
KLRG1	2F1	Biolegend
TCRβ	H57-597	BioLegend
TCRγδ	GL3	BioLegend
ST2	DIH9	BioLegend
IL-5	TRFK5	BioLegend
IL-17A	TC11-18H10.1	BioLegend
Ly6G	1A8	BD Biosciences
Siglec-F	E50-2440	BD Biosciences
F4/80	BM8	ThermoFisher
IL-13	eBio13A	ThermoFisher
Ym1	Polyclonal	R&D Systems
RELM-α	Polyclonal	Peprotech
IFNγ	XMG1.2	Biolegend
CD69	H1.2F3	Biolegend
GATA3	16E10A23	Biolegend
EGFR	EGFR1	Abcam

### Quantification of cytokines

Single-cell suspensions of splenocytes, lung-draining lymph nodes or whole lung were stimulated *ex vivo* with *N. brasiliensis* excretory secretory product (E/S) antigen^41^ (1 μg/ml) or anti-CD3 (1 μg/ml). Cell supernatants were harvested 72 h later and were stored at −20 °C until further analysis. Mouse IL-13 DuoSet ELISA kit (R&D Systems) was used for measurement of IL-13 levels. Mesenteric lymph node (MLN) cells from *T. muris* infected or uninfected mice were collected, cultured and restimulated ex vivo for 36 h with E/S as previously described.^22^ The concentrations of IL-5, IL-6, IL-9, IL-10, IL-13, IL-17A, TNFα and IFNγ in the mLN culture supernatant were measured by cytokine bead array (CBA, BD Biosciences, UK) as per the manufacturer’s protocol.

### Antibody depletion experiments

IFNγ was depleted using an anti-IFNγ monoclonal antibody (clone XMG1.2) and injected intraperitoneally (500 μg/mouse/day) on days −1 and 1 of infection with *N. brasiliensis*. Control mice were injected with an equal amount of corresponding isotype control (GL113). IL-17A was depleted using an anti-IL-17A (17F3) or IgG1 isotype (both Invivo mAB) injected intraperitoneally (100 μg/mouse/day) on days 4, 5 and 6 post-infection with *N. brasiliensis*.

### Extraction of RNA and quantitative real-time PCR

A fragment of the right lung lobe was stored in RNAlater (Ambion) before homogenization of tissue in Qiazol reagent with a TissueLyser (Qiagen). RNA was prepared according to manufacturer’s instructions. RNA was quantified using a ND-1000 Spectrophotometer (NanoDrop Technologies). Reverse transcription of 1 μg of total RNA was performed using Tetro reverse transcriptase (Bioline). For reverse transcription, total RNA was treated with 50 U Tetro reverse transcriptase (Bioline), 40 mM dNTPs (Promega), 0.5 μg PolyT primer for cDNA synthesis (Roche) and RNasin inhibitor (Promega). The abundance of transcripts from the genes of interest was measured by quantitative real-time PCR with the Light Cycler 480 II system (Roche) with a Brilliant III SYBR master mix (Agilent) and specific primer pairs. PCR amplification was analysed by the second-derivative maximum algorithm (Light Cycler 480 Sw 1.5; Roche), and expression of the gene of interest was normalized to that of the housekeeping gene *Actb* (beta-actin). A list of primer sequences used are shown in Table 2.

**Table 2:** List of primer sequences used.

Primer	Sequence (5′-3′)
*Ccl8* forward	TTCTTTGCCTGCTGCTCATA
*Ccl8* reverse	AGCAGGTGACTGGAGCCTTA
*Il5* forward	ACATTGACCGCCAAAAAGAG
*Il5* reverse	CACCATGGAGCAGCTCAG
*Chil3* forward	ACCTGCCCCGTTCAGTGCCAT
*Chil3* reverse	CCTTGGAATGTCTTTCTCCACAG
*Il4* forward	CCTGCTCTTCTTTCTCGAATGT
*Il4* reverse	CACATCCATCTCCGTGCAT
*Retnla* forward	TATGAACAGATGGGCCTCCT
*Retnla* reverse	GGCAGTTGCAAGTATCTCCAC
*Il13* forward	CGTTGCACAGGGGAGTCT
*Il13* reverse	CCTCTGACCCTTAAGGAGCTTAT
*Ifng* forward	GGAGGAACTGGCAAAAGGAT
*Ifng* reverse	TTCAAGACTTCAAAGAGTCTGAGG
*Actb* forward	GCCGGACTCATCGTACTCC
*Actb* reverse	GTGACGTTGACATCCGTAAAG
*Muc5ac* forward	GCATCAATCAACAGCGAAACTT
*Muc5ac* reverse	CGAGTCACCCCCTGAGTC
*Muc5b* forward	GAGGTCAACATCACCTTCTGC
*Muc5b* reverse	TCTCATGGTCAGTTGTGCAGG

### *Trichuris muris* infection and E/S products

*T. muris* eggs were prepared from chronically infected stock mice as described previously.^42^ Mice were infected by oral gavage with 200 embryonated *T. muris* eggs suspended in ddH2O. At day 19 and 32 post infection, *T. muris* burden was assessed by removing the caecum and proximal colon, opening them longitudinally and scraping the contents out with fine forceps. Individual worms were then counted by eye under a binocular dissecting microscope. *T. muris* adult excretory secretory product antigen (E/S) was prepared as described by.^42^ In brief, adult *T. muris* were cultured ex vivo at 37 °C, the culture supernatant was collected and centrifuged to remove eggs and worms. The resultant supernatant was then filter sterilised and stored at −20 °C until use for in vitro re-stimulation of MLN cells.

### Nanostring RNA profiling

Extracted RNA was run on an Agilent 2200 Tape Station system to ensure high quality lung RNA; samples with a RIN value of <6.5 were excluded. Suitable RNA was then diluted to 20 ng/μL in RNase free H2O, measured using Qubit™ RNA HS Assay Kit (Thermofisher) and run on a Nanostring nCounter^®^ FLEX system using the Myeloid Innate Immunity v2 panel (XT-CSO-MMII2-12) 220 as per manufacturer’s instructions. Raw data were loaded into nSolver version 4.0 using default settings. Non-normalised counts were then exported and subsequent analyses were performed in R (version 3.6) using RStudio Version 1.2.1335 Build 1379 – © 2009–2019 RStudio, Inc. Positive controls were analysed to ensure there was clear resolution at variable expression levels and negative controls were used to set a minimum detection threshold which was applied to all samples. Data were then normalised with EdgeR using the TMM method and differential expression between *N. brasiliensis*-infected WT and *Il17a*-KO mice was calculated via linear modelling with Empirical Bayes smoothing using the limma R package 2.^43^ Genes with an absolute fold change of greater than one and a significance value of under 0.05 after correction for multiple comparisons using the Benjamini-Yekeuteli method were defined as “differentially expressed” and taken forward for further analysis. Heatmaps were then generated from normalized counts of DE genes using the ComplexHeatmaps R package. The networks and functional analyses of DE genes were generated with Ingenuity pathway analyser (QIAGEN Inc., https://www.qiagenbio-informatics.com/products/ingenuity-pathway-analysis). Data were then imported into R for visualisation.

### Statistics

Prism 7.0 (version 7.0c, GraphPad Software) was used for statistical analysis. Differences between experimental groups were assessed by ANOVA (for normally distributed data, tested using Shapiro-Wilk normality test) followed by Sidak’s multiple comparisons test. For gene expression data, values were log_2_ transformed to achieve normal distribution. Comparisons with a *P* value < 0.05 were considered to be statistically significant. Data are represented as mean ± sem.

## Supplementary information

Supplementary Figures
