# Supplementary figures and images for: IL-17A both initiates, via IFNγ suppression, and limits the pulmonary type-2 immune response to nematode infection
Source: Mucosal Immunol. 2020 Jul 7;13(6):958–68. doi: 10.1038/s41385-020-0318-2 (PMC7567645; doi:10.1038/s41385-020-0318-2)

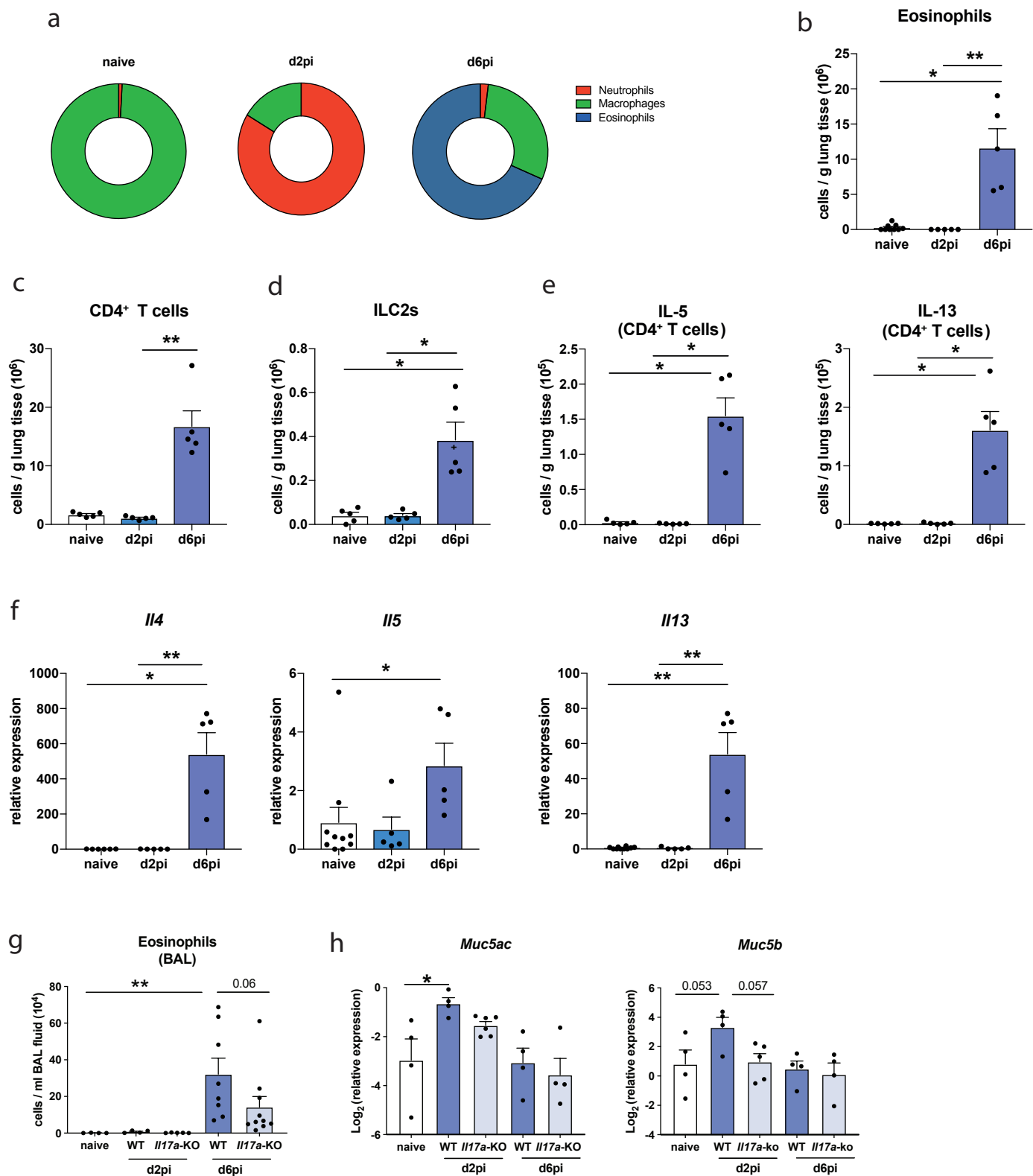

Supplement figure 1

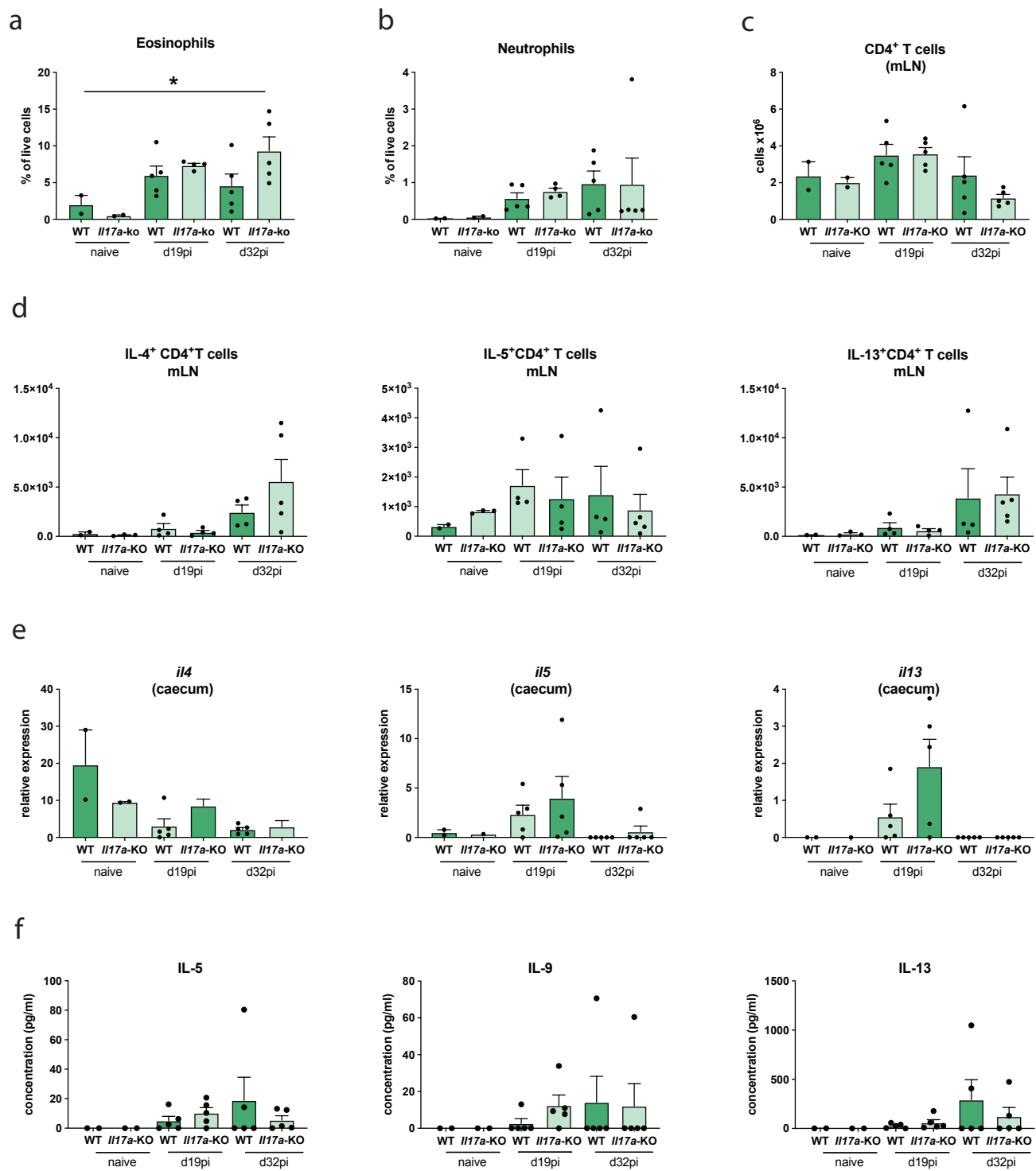

Supplement figure 2

Supplement: Supplementary file 1 — Supplementary Figures [file 41385_2020_318_MOESM1_ESM.pdf]
